# Evaluation of Increasing Concentrations of Supplemental Choline Chloride on Modern Broiler Chicken Growth Performance and Carcass Characteristics

**DOI:** 10.3390/ani13091445

**Published:** 2023-04-23

**Authors:** Caroline R. Gregg, Brittany L. Hutson, Joshua J. Flees, Zachary S. Lowman, Kari A. Estes, Jessica D. Starkey, Charles W. Starkey

**Affiliations:** 1 Department of Poultry Science, Auburn University, Auburn, AL 36832, USA; 2 Animal Nutrition and Health Division, Balchem Corporation, New Hampton, NY 10958, USA; 3 North American Renderers Association, Alexandria, VA 22314, USA

**Keywords:** broiler chicken, choline chloride, growth performance, carcass yield, wooden breast

## Abstract

**Simple Summary:**

Broilers were fed increasing levels of supplemental choline chloride in diets where methionine was minimally reduced (0.15%), reared under summer conditions for 41 days, and then processed. Supplemental choline improved broiler feed efficiency by reducing feed intake without altering body weight gain. Increasing dietary choline concentrations also increased carcass yield, breast yield, and the incidence and severity of wooden breast. It can be concluded that when broilers are reared under high environmental temperature and fed diets with reduced methionine, supplemental choline chloride can positively impact growth performance and carcass yields.

**Abstract:**

Choline has been demonstrated to partially substitute methionine in broiler chicken diets due to their interconnected biosynthesis pathways. Yet, research on the impacts of dietary choline supplementation on modern strains of high-yielding broilers is limited. The objective was to evaluate the effect of increasing additions of choline chloride on the performance and carcass characteristics of broilers fed reduced methionine diets and reared under summer environmental conditions. Ross 708 x Yield Plus male broilers were reared for 41 days on used litter in floor pens (n = 2232; 31 birds per pen). Birds were fed one of six corn and soybean meal-based, reduced methionine diets containing 0, 400, 800, 1200, 1600, or 2000 mg of added choline chloride per kg of feed. Diets were provided in three phases. On day 43, 10 birds per pen were processed. Increasing dietary choline resulted in similar body weight gain, reduced feed intake, and improved feed efficiency. Choline chloride supplementation linearly increased both breast and carcass yields while concomitantly increasing the incidence and severity of wooden-breast-affected fillets. These results indicate that supplementing reduced-methionine broiler diets with choline chloride during high environmental temperatures may improve feed efficiency and increase carcass and breast yields but may also increase wooden breast.

## 1. Introduction

Choline is a nutrient similar to an amino acid that functions as an important precursor for cell membrane lipids, neurotransmitters, and methylated compounds. Its role as a methyl donor in the biosynthesis of compounds containing methyl groups is important for the production and availability of methionine (Met) [1,2]. Choline enters the s-adenosyl methionine (SAM) pathway when irreversibly oxidized to betaine. The methyl groups in betaine are then passed to a derivative of folic acid that is later converted to Met. SAM is formed from Met and acts as a high-energy methyl donor that is important for many anabolic reactions. In cases where choline is insufficient to fuel the regeneration of Met, Met is recovered from protein breakdown in the body [3].

Choline can be synthesized de novo from ethanolamine in all nucleated animal cells, specifically in the liver where phosphatidyl choline is needed for lipoprotein synthesis. [4] However, de novo choline synthesis alone does not appear to be sufficient, and choline concentration in the body depends heavily on the presence of adequate Met, betaine, and folic acid [5,6]. For this reason, it is difficult to assess dietary choline requirements without also controlling SAM pathway intermediates.

Animals rely on dietary sources of preformed choline to make up for poor de novo synthesis. Fatty liver development that is caused by a deficiency in dietary choline [7] has been implicated as a potential way that inadequate choline impacts the performance of production poultry as choline plays a role in lipid transport and metabolism [8,9]. Reduced fat content in the liver has been observed in laying hens and broiler breeders fed supplementary choline [10,11]; however, additional choline did not alter the egg production or feed efficiency of broiler breeders. Supplementing 1200 mg of dietary choline per kg of feed to Ross 308 broiler chicks caused an increase in feed intake but no change to feed conversion when dietary Met concentrations were above broiler nutrient recommendations [12]. Jahanian & Ashnagar [13] reported reduced feed intake and improved FCR in Ross 308 broilers fed additional choline.

Heat stress is a common issue facing the broiler industry, specifically during the summer months in the Southeastern United States. Heat stress negatively impacts growth performance, increases mortality, and can alter carcass characteristics [14,15]. Betaine, which can be directly oxidized from choline, is thought to protect against heat stress due to its osmoregulatory qualities that can help decrease cellular dehydration [16]. Mahmoudi et al. [17] evaluated the effects of replacing dietary Met with choline and betaine in Ross 308 broilers exposed to heat stress. These authors did not observe differences in average daily feed intake or body weight gain in birds fed either choline, betaine, or a combination of the two, thus suggesting that Met can be replaced with choline and betaine in heat-stressed broilers. While it has been observed that supplementing broiler diets with betaine is more efficient than choline at increasing betaine concentrations in the liver where choline is converted to betaine [18], Kpodo et al. [19] reported similar plasma betaine concentrations in broilers fed diets supplemented with either choline or betaine. This suggests feeding either choline or betaine can have similar metabolic benefits as they result in comparable increases in circulating betaine.

Feeding increasing additions of choline chloride on top of the basal choline provided by a corn and soybean meal-based diet to high-yielding, large-frame Ross 708 x Yield Plus broilers did not previously result in improvements in growth or carcass traits when dietary Met was adequate and rearing temperatures were maintained according to bird comfort [20,21]. Therefore, the aim of the present study was to evaluate the effects of increasing concentrations of supplementary choline chloride on growth performance and carcass characteristics of broilers fed diets with reduced Met under summer rearing conditions.

## 2. Materials and Methods

All procedures involving live birds were reviewed and approved by the Auburn University Institutional Animal Care and Use Committee (PRN 2020-3749).

### 2.1. Diet Formulation

Six experimental corn and soybean meal-based diets were manufactured in the Auburn University Poultry and Animal Nutrition Center Feed Mill. Diets were formulated according to 2019 Aviagen Nutrition Specifications [22] with the exception of a 15% reduction in digestible Met. Choline chloride was supplemented at either 0, 400, 800, 1200, 1600, or 2000 mg per kg of feed on top of the intrinsic concentration of choline provided by the ingredients of the basal diet as used in previous experiments [20,21]. Diets were fed in 3 phases: crumbled starter from d 0 to d 15, pelleted grower from d 16 to d 28, and pelleted finisher from d 29 to d 41 (Table 1). All diets were third-party tested for total choline content using ion chromatography (Table 2; Eurofins Nutrition Analysis Center, Des Moines, IA, USA).

### 2.2. Broiler Husbandry

Day-old Ross 708 x Yield Plus male broiler chicks were transported to the Auburn University Charles C. Miller, Jr. Poultry Research and Education Center in Auburn, AL. Chicks were randomly allotted into 72 pens (12 replicates per diet; n = 31 birds per pen), and pen weights were obtained. Chicks were placed into floor pens (0.075 m^2^ per bird) on used litter in a plenum-style poultry house. Both water and feed were provided ad libitum. Ambient temperature set points started at 34.4 °C at the time of chick placement and decreased in temperature to reach a final set point of 26.7 °C on day 21 through the remainder of the study. Birds received 23 h of light from 0 to 3 days of age, 20 h of light from 4 to 7 days of age, and 16 h of light from day 8 onwards. Light intensity was set to 30 lux from 0 to 7 days of age, 10 lux from 8 to 14 days of age, and 5 lux from day 15 through the end of the study.

### 2.3. Broiler Growth Performance and Carcass Part Yields

Mortality-corrected feed intake (FI), body weight gain (BWG), and feed conversion ratio (FCR; FI:BWG) were calculated using pen weights on day 0 and individual bird body weights on days 15, 28, and 41. Mortality was recorded on a twice daily basis to account for body weight gained by birds that died before the end of a feeding phase. On day 43, 720 birds (n = 10 birds per pen) were processed at the Auburn University Fortenberry Poultry Processing Plant following an 8-hour fast. Birds were selected for processing based on proximity to the median individual day 41 body weight of each pen. Individual fasted, live body weights of selected birds were recorded prior to slaughter, and hot carcass weights were recorded immediately following evisceration. Carcasses were then chilled in a static water bath for 3 h, and cold carcass weights as well as abdominal fat pad weights were determined. Carcasses were deboned the next day by professional commercial deboners. Carcass parts were separated, and weights of the following parts were recorded: boneless and skinless breasts (*Pectoralis major*) without rib meat, tenders (*Pectoralis minor*), bone-in and skin-on wings, bone-in and skin-on drumsticks, and bone-in and skin-on thighs. Hot carcass yield was calculated as a proportion of the fasted, live body weight, and carcass part yields were calculated as proportions of the cold carcass weight.

### 2.4. Wooden Breast and White Striping Scoring

Breast fillets were assigned Wooden Breast (WB) and White Striping (WS) severity scores using a 4-point scale (0 = normal, 1 = mild, 2 = moderate, and 3 = severe). WB was evaluated based on the degree of palpable hardness of each fillet using a tactile examination of both sides of the deboned breast. Visual evaluation of WS was conducted concurrently to ascertain the proportion of both sides of the breast containing white striations as previously described [23]. Fillets with a score of 0 were free of defects and considered normal, fillets with a score of 1 were up to ¼ affected, fillets with a score of 2 were up to ½ affected, and fillets with a score of 3 were greater than ½ affected. All fillets were scored by one pre-trained evaluator. Scores for both WB and WS are presented as a proportion of all breasts within the treatment group and as a mean score for each added choline chloride treatment.

### 2.5. Statistical Analysis

A randomized, complete block experiment with 12 replicate pens per treatment was designed. Replicate pens represented each of the 6 dietary treatments (0, 400, 800, 1200, 1600, and 2000 mg of added choline chloride per kg of feed). The GLIMMIX procedure of SAS software, ver. 9.4, was employed to analyze all data as a one-way ANOVA. Polynomial orthogonal contrasts were also evaluated. The pen served as the experimental unit with location as the blocking factor. The fixed effect was the supplemental choline chloride dietary treatment, and degrees of freedom were corrected using the Satterthwaite adjustment. The events/possible events syntax with a binomial distribution and R-side covariance structure in SAS was used to evaluate proportional data (e.g., mortality and carcass yields). The PDIFF option was used to conduct all least squared means separations; means were declared different when *p* < 0.05, and tendencies were declared when 0.05 < *p* ≤ 0.10.

## 3. Results

### 3.1. Growth Performance

As shown in Table 3, supplementing broiler diets with choline resulted in improved feed intake during the finisher and overall growth period. This change in feed intake was observed without altering BWG. Changes in FCR do not appear to follow a specific trend across treatments during the starter and grower phase. However, FCR was reduced in broilers fed 800 or more mg of added choline chloride per kg of feed during the finisher phase. Orthogonal contrasts revealed that the reduction in FCR as choline supplementation increases overall from day 0 to 41 of age was linear (*p* < 0.0001). Mortality was not impacted by dietary choline supplementation (Table 4).

### 3.2. Carcass Characteristics

Table 5 reports data obtained from processing at day 43 of age. Hot Carcass HC yield was calculated as a proportion of d 43 fasted live body weight (FLBW). HC yield increased as choline supplementation increased. Dietary treatments of 1200 or more mg of additional choline chloride per kg of feed resulted in the heaviest breast weights and greatest breast yields, thus generating a 78 g improvement when comparing the control with the 2000 mg. Conversely, supplementing 2000 mg of choline chloride decreased abdominal fat pad yield compared with birds fed no additional choline. Similar effects were observed with the reduction in wing, thigh, and drumstick yields as dietary choline increased.

### 3.3. Wooden Breast and White Striping

The incidence and severity of WB and WS myopathies were assessed and displayed in Table 5. The incidence of severe WB, or breasts assigned a score of 3, increased as supplementary choline concentrations increased. A greater number of breasts from birds not fed additional choline were assigned a score of 0 and lacked signs of WB development than all other groups. This follows a similar pattern to breast yield. The incidence of severe WB appears to increase as breast yield increases. Average WB scores also increased with supplemental choline inclusions in the diet; however, average WB scores were below 2 for all treatments. The incidence of WS score 3 tended to increase as supplemental choline increased. Otherwise, no differences were observed in the incidence or severity of WS among dietary treatments.

## 4. Discussion

Adding supplemental choline to broiler diets may spare low dietary concentrations of Met as shown by the observed improvements in feed efficiency as choline concentration increased without altering BW gain. This is in agreement with a previous study that found supplementing choline in a Met-deficient diet results in a similar growth performance to broilers fed sufficient concentrations of Met [17]. The combination of reduced Met and high-temperature conditions likely amplified the positive impacts of dietary choline on feed efficiency as Gregg et al. [20,21] did not observe changes in growth performance of broilers fed increasing concentrations of choline chloride when dietary Met was sufficient and rearing temperature was optimal. However, supplementation of dietary choline has not been previously demonstrated to alleviate losses in body weight gain associated with heat stress [24]. This suggests that the Met sparing effect of dietary choline may have more of an impact on broiler performance in the present study than any potential impacts choline has on growth during heat stress.

The observed increase in breast yield combined with a reduction in wing, thigh, drumstick, and abdominal fat yield suggests that additional dietary choline may aid in diverting more nutrients to the growth of lean muscle than the less-desirable carcass parts and adipose tissue. This is similar to results reported by Waldroup et al. [25] who observed improvements in breast yield with added dietary choline, but did not observe an interaction between choline and Met concentrations in the diet. Jahanian & Ashnagar [13] studied the lipotropic effects of adding choline to broiler diets. These authors reported an increase in carcass yield and a reduction in abdominal fat in birds fed additional choline. Interestingly, they also noted a reduction in analyzed fat content of the breast muscle in choline-supplemented broilers. This is consistent with the reduction in abdominal fat pad yield observed in broilers supplemented with the greatest concentration of choline in the present study. In swine, dietary choline was found to improve fatty acid oxidation and reduce circulating free fatty acids and triglycerides [26]. Therefore, additional dietary choline may promote leaner broiler carcasses due to its role in lipid metabolism.

The greatest increases in both WB severity and breast yield occurred with the supplementation of an additional 1200 or greater mg of choline chloride per kg of feed. This increase in WB incidence and severity is consistent with current knowledge of WB as the development of the myopathy strongly correlates with increased growth rate and breast yields [27,28]. Therefore, it is likely that the observed increases in WB incidence can be attributed to improvements in breast yield as there is no known mechanism for the role of choline in the development of WB. Increasing levels of dietary choline appear to protect against a reduction in feed efficiency caused by high environmental temperatures utilized in this experiment. Heat stress is understood to result in diminished growth performance in broilers [14]. Previous literature shows that decreases in WB incidence in heat-stressed birds is related to a reduction in growth performance and breast yield [29,30]. Based on the results of the present study, increasing additions of dietary choline may counteract the impacts of heat stress on growth performance, specifically reduced feed intake and poor feed efficiency. This would negate the reduction in WB commonly observed under heat stress conditions, therefore helping to explain the increase in WB incidence with increased choline when reared under high-temperature conditions.

## 5. Conclusions

Feeding additional choline to high-yielding broilers improved overall feed efficiency, and supplementing 1200 or more mg of choline chloride per kg of feed increased carcass and breast yields when Met was insufficient. This evidence supports the supplementation of choline chloride in high-yielding broiler diets where Met is limited or as a partial substitute for Met. The observed improvements in breast yield resulting from choline supplementation appear to lead to an increase in WB severity. Given the osmoregulatory qualities of betaine and its ability to be oxidized to choline, dietary choline supplementation may also be beneficial in improving growth efficiency in broilers reared during summer months when heat stress is common.

## Figures and Tables

**Table 1:** Ingredients and nutrient composition of basal broiler chicken diets.

Ingredients, % (As Fed) ^1^	Feeding Phase		
	Starter, d 0 to 15	Grower, d 16 to 28	Finisher, d 29 to 41
Corn	72.34	67.45	68.17
SBM	22.00	25.00	23.00
DDGS	--	2.00	4.00
Soybean oil	0.10	0.75	0.75
Dicalcium phosphate	2.36	1.94	1.61
Calcium carbonate	0.90	0.82	0.77
Sodium chloride	0.40	0.40	0.35
L-Lysine hydrochloride	0.59	0.52	0.33
DL-Methionine	0.19	0.14	0.10
L-Threonine	0.42	0.29	0.21
Mineral premix	0.10	0.10	0.10
Vitamin premix	0.10	0.10	0.10
Choline chloride premix ^2^	0.50	0.50	0.50
Calculated Nutrient Content of Basal Diet			
Choline ion, mg per kg	985.19	1065.76	1065.962
Betaine, mg per kg	39.98	44.68	46.80
Digestible Methionine, %	0.42	0.39	0.35
Digestible Lysine, %	1.26	1.26	1.04
Crude protein, %	16.07	17.65	17.12
Calcium, %	0.95	0.84	0.75
Available phosphorus, %	0.48	0.42	0.37
AME_n_, kcal per kg	2860.00	2849.74	2870.01
Analyzed Nutrient Content of Basal Diet			
Methionine, %	0.42	0.42	0.37
Choline ion, mg per kg	910.00	977.00	987.00
Betaine, mg per kg	41.73	40.13	36.70

^1^ SBM = soybean meal, DDGS = dried distillers’ grain with solubles. ^2^ Choline premix contained ground rice hulls and added choline chloride.

**Table 2:** Analyzed choline ion concentration (mg per kg) in broiler chicken diets with increasing additions of choline chloride.

Feeding Phase	Dietary Treatment					
	Analyzed Dietary Choline Ion Values, mg per kg of Feed (as Fed)					
	0	400	800	1200	1600	2000
Starter, d 0 to 15	910	1120	1400	1650	1950	2330
Grower, d 16 to 28	977	1190	1500	1750	2050	2380
Finisher, d 29 to 41	987	1230	1550	1880	2060	2270

**Table 3:** Effect of supplemental dietary choline chloride on broiler chicken growth performance from d 0 to 41 ^1^.

Variable ^2^	Added Choline Chloride, mg per kg “as Fed”						SEM ^3^	*p*-Value
	0	400	800	1200	1600	2000		
d 0 BW, g	41	41	41	42	41	41	0.2	0.3505
d 15 BW, g	394 ^x^	380 ^y^	387 ^xy^	377 ^y^	378 ^y^	385 ^xy^	5	0.0824
d 28 BW, g	1295	1279	1273	1266	1256	1273	11	0.2049
d 41 BW, g	2511	2491	2518	2491	2509	2507	23	0.9349
d 0 to 15 MCBWG, g	351 ^x^	337 ^y^	343 ^xy^	333 ^y^	334 ^y^	342 ^xy^	5	0.0543
d 16 to 28 MCBWG, g	901	897	886	882	878	888	7	0.2186
d 29 to 41 MCBWG, g	1215	1209	1244	1234	1253	1255	16	0.2305
d 0 to 41 MCBWG, g	2444	2411	2438	2416	2424	2461	25	0.7408
d 0 to 15 MCFI, g	510 ^a^	489 ^b^	494 ^b^	493 ^b^	489 ^b^	489 ^b^	5	0.0115
d 16 to 28 MCFI, g	1324 ^x^	1303 ^xy^	1315 ^x^	1296 ^xy^	1281 ^y^	1296 ^xy^	11	0.0830
d 29 to 41 MCFI, g	2175 ^a^	2135 ^ab^	2142 ^ab^	2116 ^b^	2119 ^b^	2109 ^b^	15	0.0229
d 0 to 41 MCFI, g	3995 ^a^	3908 ^b^	3925 ^ab^	3871 ^b^	3862 ^b^	3896 ^b^	29	0.0217
d 0 to 15 MCFCR	1.4533 ^abc^	1.4550 ^abc^	1.4383 ^bc^	1.4836 ^a^	1.4633 ^ab^	1.4333 ^c^	0.01	0.0220
d 16 to 28 MCFCR	1.4700 ^b^	1.4517 ^c^	1.4850 ^a^	1.4600 ^bc^	1.4655 ^b^	1.4592 ^bc^	0.01	<0.0001
d 29 to 41 MCFCR	1.7908 ^a^	1.7667 ^a^	1.7233 ^b^	1.7175 ^b^	1.6917 ^b^	1.6950 ^b^	0.01	<0.0001
d 0 to 41 MCFCR	1.6309 ^a^	1.6217 ^a^	1.6100 ^ab^	1.5936 b^c^	1.5942 b^c^	1.5850 ^c^	0.01	0.0017

^1^ Each dietary treatment consisted of 12 replicate pens (31 birds per pen). Broilers received diets provided in three phases: starter (d 0 to 15), grower (d 16 to 28), and finisher (d 29 to 41). Pen means above and below 3 standard deviations of the population mean for each variable were removed. ^2^ MC = mortality-corrected; BW = body weight; BWG = body weight gain; FI = feed intake; FCR = feed conversion ratio (feed:gain). ^3^ SEM = highest standard error of the least squared mean comparisons. ^abc^ Means within in a row with different superscripts differ *p* ≤ 0.05. ^xy^ Means within a row with different superscripts differ 0.0501 ≤ *p* ≤ 0.10 and are considered tendencies.

**Table 4:** Effect of supplemental dietary choline chloride on broiler chicken mortality from d 0 to 41 ^1^.

Variable	Added Choline Chloride, mg per kg of Feed						SEM ^2^	*p*-Value
	0	400	800	1200	1600	2000		
d 0 to 15 Mortality, %	1.08	2.42	2.15	1.34	2.15	1.61	0.83	0.7548
d 16 to 28 Mortality, %	1.09	0.28	0.46	0.53	0.38	0.27	0.53	0.6220
d 29 to 41 Mortality, %	0.55	1.66	1.39	1.10	1.66	1.10	0.64	0.8019
d 0 to 41 Mortality, %	2.69	4.30	4.30	3.50	4.30	2.96	1.10	0.7657

^1^ Each dietary treatment consisted of 12 replicate pens (31 birds/pen). Broilers received diets provided in three phases: starter (d 0 to 15), grower (d 16 to 28), and finisher (d 29 to 41). ^2^ SEM = highest standard error of the least squared mean comparisons.

**Table 5:** Effect of supplemental dietary choline chloride on carcass characteristics and yield of 43-day-old broiler chickens ^1^.

Variable ^2^	Added Choline Chloride, mg per kg of Feed						SEM ^3^	*p*-Value
	0	400	800	1200	1600	2000		
Hot WOG WT, g	1912 ^y^	1919 ^y^	1950 ^xy^	1945 ^xy^	1957 ^xy^	1978 ^x^	17	0.0735
Chilled WOG WT, g	1940 ^y^	1947 ^y^	1977 ^xy^	1974 ^xy^	1985 ^xy^	2006 ^x^	17	0.0681
Fat Pad WT, g	29	27	29	27	27	27	1	0.1394
Breast WT, g	475 ^c^	502 ^b^	519 ^b^	548 ^a^	548 ^a^	553 ^a^	8	<0.0001
Tender WT, g	104	107	107	107	107	107	1	0.4928
Wing WT, g	213	212	212	210	210	212	2	0.6100
Thigh WT, g	346 ^a^	337 ^b^	336 ^b^	326 ^bc^	329 ^bc^	332 ^c^	3	0.0002
Drumstick WT, g	266 ^a^	259 ^b^	258 ^bc^	252 ^c^	255 ^bc^	255 ^bc^	2	0.0009
Hot WOG, % of FLBW	73.36 e	73.83 ^d^	74.60 ^c^	75.05 ^ab^	74.93 ^b^	75.26 ^a^	0.11	<0.0001
Hot WOG, % of chilled WOG	98.57	98.58	98.63	98.57	98.56	98.59	0.07	0.9799
Fat Pad, % of chilled WOG	1.48 ^a^	1.4 ^ab^	1.44 ^ab^	1.35 ^bc^	1.38 ^abc^	1.33 ^c^	0.04	0.0293
Breast, % of chilled WOG	24.48 ^c^	25.76 ^b^	26.24 ^b^	27.76 ^a^	27.62 ^a^	27.59 ^a^	0.26	<0.0001
Tender, % of chilled WOG	5.34	5.50	5.40	5.41	5.40	5.34	0.06	0.4665
Wing, % of chilled WOG	10.99 ^a^	10.89 ^ab^	10.72 ^bc^	10.63 ^cd^	10.54 ^d^	10.57 ^cd^	0.07	<0.0001
Thigh, % of chilled WOG	17.82 ^a^	17.33 ^b^	17.00 ^c^	16.52 ^d^	16.58 ^d^	16.54 ^d^	0.09	<0.0001
Drumstick, % of chilled WOG	13.75 ^a^	13.30 ^b^	13.04 ^c^	12.79 ^d^	12.83 ^cd^	12.69 ^d^	0.09	<0.0001
Wooden Breast score 0, %	36.75 ^a^	21.01 ^b^	17.39 ^b^	9.40 ^b^	11.02 ^b^	9.48 ^b^	5.83	0.0009
Wooden Breast score 1, %	42.79 ^ab^	47.9 ^a^	41.74 ^ab^	29.91 ^bc^	27.12 ^c^	30.17 ^bc^	4.82	0.0095
Wooden Breast score 2, %	20.51 ^y^	26.05 ^xy^	29.57 ^xy^	38.46 ^x^	37.29 ^x^	35.34 ^x^	5.22	0.0909
Wooden Breast score 3, %	0.00 ^a^	5.04 ^b^	11.30 ^b^	22.22 ^c^	24.58 ^c^	25.00 ^c^	3.74	0.0004
White Striping score 0, %	16.24	8.40	5.22	10.26	10.17	6.03	3.54	0.1121
White Striping score 1, %	53.85	52.94	51.30	51.28	52.54	52.59	4.60	0.9986
White Striping score 2, %	23.08	32.77	30.43	29.91	25.42	27.59	4.19	0.5741
White Striping score 3, %	6.84 ^z^	5.88 ^z^	13.04 ^xy^	8.55 ^xyz^	11.86 ^xyz^	13.79 ^x^	2.63	0.0763
Mean Wooden Breast score	0.91 ^d^	1.25 ^cd^	1.33 ^bc^	1.75 ^a^	1.83 ^a^	1.67 ^ab^	0.13	<0.0001
Mean White Striping score	1.08	1.42	1.58	1.25	1.50	1.50	0.14	0.1138

^1^ Total n = 720 (120 birds/treatment). Pen means above and below 3 standard deviations of the population mean for each variable were removed. ^2^ BW = body weight; FLBW = fasted live body weight; WOG = without giblets; WT = weight. ^3^ SEM = highest standard error of the least squared mean comparisons. ^abcd^ Means within in a row with different superscripts differ *p* ≤ 0.05. ^xyz^ Means within a row with different superscripts differ 0.0501 ≤ *p* ≤ 0.10 and are considered tendencies.

## Data Availability

The data presented in this study are available on request from the corresponding author.
